# Impacts of Climatic Factors on Milking Frequency and Milk Yield in Automatic Milking Systems Under Hot-Humid Conditions

**DOI:** 10.3390/ani16193106

**Published:** 2026-10-02

**Authors:** Chia-Jung Lee, Tzong-Faa Shiao, Yi-Chun Lin

**Affiliations:** 1 Livestock Management Division, Livestock Research Institute, Tainan 71246, Taiwan; shisah1218@gmail.com (C.-J.L.); tfshiao@gmail.com (T.-F.S.); 2 Department of Animal Science, National Chung Hsing University, Taichung 40227, Taiwan; 3 The iEGG and Animal Biotechnology Research Center, National Chung Hsing University, Taichung 402, Taiwan

**Keywords:** heat stress, threshold, delayed effect, supplementary feed, cows, subtropical climate, dairy, milking frequency, milk yield, automatic milking systems

## Abstract

Heat stress in hot and humid environments can severely limit the expected advantages of automatic milking systems (AMSs). This study was conducted to investigate the environmental thresholds for milking frequency (MF) and milk yield (MY) in AMSs under hot and humid conditions. We collected milking data over a 2-year period (from 29 March 2021 to 28 March 2023) from an AMS barn with an average herd size of 20.0 ± 2.2 cows (primarily Holstein cows) to investigate the effects of the temperature–humidity index (THI), dry-bulb temperature, and relative humidity on MF and MY. Both MF and MY were significantly negatively correlated with THI, temperature, and relative humidity, while a strong positive association was observed between MF and MY. Under the herd management conditions of this farm, the THI thresholds for MY and MF were 73 and 70, respectively. Beyond these thresholds, each unit increase in the THI resulted in reductions of 0.522 kg/day in MY and 0.035 times/day in MF. The Spearman correlation coefficients revealed that the negative association between MY and THI reflected a lagged, cumulative effect of weather on MY. A management-driven change in supplementary feed from soybean hulls to concentrate feed was associated with higher adjusted MF and MY. The CF period also showed higher observed THI thresholds for MF and MY than the SH period. Overall, heat stress remained a major constraint on AMS performance under hot-humid conditions, supporting the need for effective cooling and management strategies.

## Simple Summary

Automatic milking systems (AMSs) are widely adopted to reduce labor requirements on dairy farms. However, their performance depends on cows’ willingness to voluntarily visit the milking unit. In hot and humid environments, heat stress may reduce both milk yield and milking frequency, potentially limiting the expected advantages of AMSs. Using data from a subtropical dairy farm equipped with an AMS, this study examined how climatic conditions affect milking behavior and milk production and whether a management-driven change in supplementary feed was associated with differences in these responses under farm conditions. The results showed that when environmental heat load exceeded specific thresholds, both milking frequency and milk yield declined rapidly. The concentrate feed period was associated with higher milk yield and higher observed heat-stress thresholds than the soybean hull period. In hot and humid climates, AMS performance remained highly sensitive to heat stress, and additional cooling or management interventions may be required to maintain stable milk production.

## 1. Introduction

The first automatic milking system (AMS) was officially installed in the Netherlands in 1992 [1]. This system has been increasingly adopted worldwide as a strategy to reduce labor demand and improve management efficiency on dairy farms [1]. By allowing cows to voluntarily access the milking unit, AMSs can increase milking frequency (MF) and, consequently, milk yield (MY) under suitable conditions [2,3]. Previous studies have reported that higher MF in AMSs is generally associated with increased milk production, particularly during early lactation [4]. However, the performance of AMSs relies heavily on cows’ motivation and willingness to voluntarily visit the milking unit, which can be strongly influenced by environmental and management factors.

Among these factors, heat stress represents a major challenge to dairy production, especially in hot and humid regions. Heat stress occurs when these environmental factors exceed certain thresholds and elevate an animal’s body temperature beyond the normal range [5]. Heat stress can alter the feeding pattern of lactating cows [6], reducing DMI [7] and thus milk production [8]. Cows under heat stress produce less milk than normal and have poorer reproduction, nutrition, and health [9]. Notably, dairy cows with high milk yield are more significantly affected by heat stress than are those with moderate to low MY [10]. Moreover, in AMSs, heat stress may further reduce voluntary milking visits, thereby limiting the expected advantages of automation. Although several studies have investigated the effects of heat stress on milk yield and milking behavior, most have been conducted under temperate climates, pasture-based systems, or conventional milking systems. Information regarding the response of AMS-managed dairy cows to heat stress under hot-humid subtropical conditions remains limited.

Environmental heat load is commonly assessed using the temperature–humidity index (THI), which integrates ambient temperature and relative humidity into a single indicator [5]. In addition to THI, individual climatic variables such as dry-bulb temperature (T_db_) and relative humidity (RH) may independently influence cow behavior and productivity [11]. Importantly, animal responses to environmental stressors are often non-linear [12], and production losses may occur only after specific environmental thresholds are exceeded [13]. Identifying such threshold values is therefore critical for developing practical management strategies to mitigate heat stress in dairy systems [14]. However, threshold-based analyses focusing simultaneously on milking frequency and milk yield in AMSs under hot-humid conditions are scarce.

In AMSs, supplementary feed provided during milking serves not only as a nutritional input but also as a behavioral incentive that encourages cows to voluntarily visit the milking unit. Furthermore, supplementary feeding increases milk production due to oxytocin stimulation [15]. Changes in supplement type or composition may therefore influence milking frequency and milk yield [16,17], particularly under challenging environmental conditions. On commercial farms, such changes often arise from routine management decisions rather than experimental design, creating opportunities for observational comparisons under real-world conditions. Understanding how different supplementation strategies interact with environmental stress may provide valuable insights for AMS management, even when causal inference is limited.

To our knowledge, few studies have simultaneously identified farm-level thresholds for both milking frequency and milk yield in indoor AMS herds under subtropical hot-humid conditions, and evidence on lagged THI effects in this setting remains limited. Therefore, the objectives of this study were as follows: (1) to evaluate the effects of specific climatic factors, namely dry-bulb temperature, relative humidity, and the temperature–humidity index, on milking frequency and milk yield in dairy cows managed under an AMS in a hot-humid environment; (2) to identify environmental threshold values beyond which MF and MY decline; and (3) to assess, under observational farm conditions, whether a management-driven change in supplementary feed from soybean hulls (SH) to concentrate feed (CF) was associated with differences in milking behavior and milk production. Specifically, we hypothesized that the negative impacts of heat stress on MF and MY follow threshold-dependent and lagged patterns, and that CF would be associated with improved voluntary milking visits and yield maintenance under hot-humid conditions. By addressing these objectives, this study aims to provide practical, threshold-based information to support AMS management decisions in hot and humid regions.

## 2. Materials and Methods

The present study was conducted at the Livestock Research Institute, Taiwan (23.049° N, 120.336° E). This work was an observational study based on routinely recorded farm data. No experimental intervention was imposed for the purpose of this study. Therefore, results should be interpreted as associations observed under commercial management conditions. Internal records from the National Agricultural Meteorological Monitoring System indicated that the average annual temperature in the study area for the period 29 March 2021 to 28 March 2023 was 24.3 °C. The highest and lowest monthly average temperatures were 29.1 °C and 16.3 °C, respectively. The average RH was 81.1%, and the average THI was 73.8.

### 2.1. Animal Management

**Figure 1:**
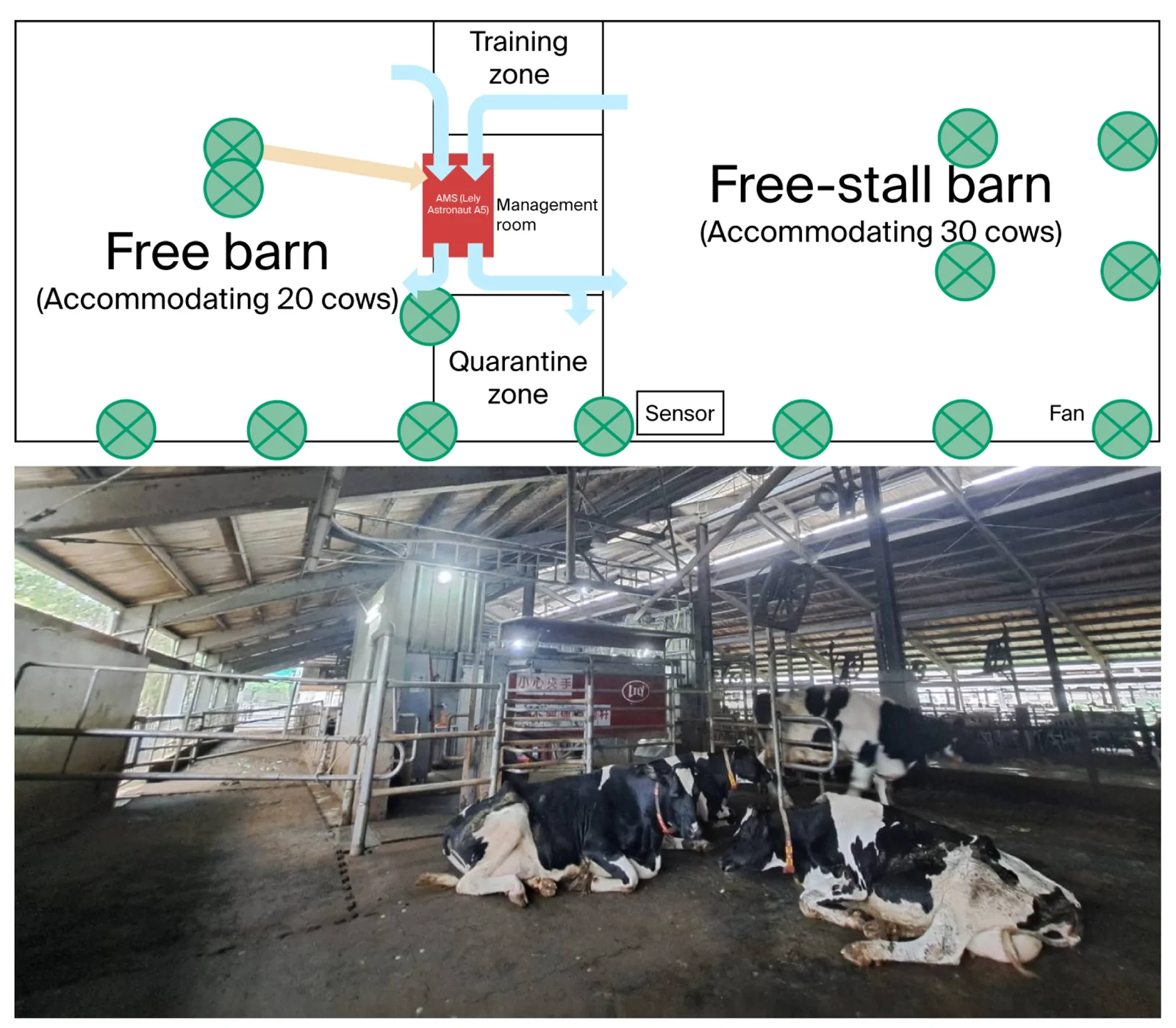
Layout and photographic view of the study barn. (**Top**) Floor plan of the barn layout, comprising a free barn and a free-stall barn, equipped with the automatic milking system (AMS) operating under a free cow traffic system. The blue arrows indicate the cow traffic routes, and the orange arrow indicates the airflow direction of the dedicated AMS fan. (**Bottom**) A partial view of the free barn and the entrance/exit area of the AMS.

Cattle were housed in a conventional barn divided into two sections, with the AMS unit located between these sections. The primary section operated as a free-stall system (accommodating 30 cows; Figure 1), whereas the secondary section operated as a loose housing system (accommodating 20 cows). At full capacity, each section provided average activity space of 10 m^2^ per cow alongside a treatment area for five cows and a waiting area for four cows. The barn operated under a free cow traffic system, allowing all cows to visit the AMS freely, except during routine cleaning and milk tank collection. The maximum distance the cows had to walk was approximately 30 m.

To mitigate heat stress, twelve 40-inch industrial fans were placed 2.8 m above the feed bunk and lying areas. These fans operated automatically when the temperature exceeded 18 °C. During summer, the average WS at a height of 1.6 m was 3.4 ± 1.6 m/s. A dedicated fan for the AMS parlor was placed 5 m from the cows at a 45° angle toward the parlor. Sprinkler and mist systems were installed above the feed bunk. These systems were automatically activated at temperatures of 28 °C and 30 °C, respectively. The sprinkler system sprayed water for 1 min at 10 min intervals, whereas the mist system sprayed mist for 30 s at 5 min intervals. The systems ceased operation when the RH reached 80–90% or after 6 PM to avoid excessive humidity.

Transitioning the cows from a conventional milking system (CMS; with tandem milking parlor) to the AMS required 5–10 days of training. During this training period, cows were guided to enter the AMS at least twice daily until they voluntarily visited the system for three consecutive days.

Under routine management, the maximum allowed MF was five milkings per day, and cows in the late lactation stage were milked a maximum of two times daily before the dry period. In order to prevent mastitis caused by prolonged milking intervals, the farm staff checked the herd three times daily (at 05:00, 08:00–10:00, and 13:00–15:00 h) and manually guided cows to the AMS if their milking interval exceeded 10 h. Outside these management hours, cows were allowed to access the AMS voluntarily without human intervention.

### 2.2. Feeding Management and Supplementary Feed Periods

Cows were fed a total mixed ration (TMR) and received supplementary feed allocated within the AMS as an incentive to enter the unit. During the observation period, supplementary feed type changed due to routine feed inventory and farm management constraints rather than an experimentally designed intervention. Specifically, soybean hulls (SH) were used initially and later replaced by concentrate feed (CF) from 25 March 2022 (Table 1). As per the farm’s routine protocol, supplementary feed was intended as a milking reward without significantly affecting the basal TMR intake. During the period, supplementation was allocated based on MY as follows: 2 kg for >45 kg/day, 1.5 kg for 35.1–45 kg /day, 1 kg for 25.1–35 kg /day, and 0.5 kg for ≤25 kg /day.

For the supplement comparison, the dataset was separated into the SH period and the CF period based on the date of the management-driven feed replacement. Throughout the data collection period, the nutritional proportions of the TMR formulation, such as crude protein and crude fiber, were not altered; however, minor adjustments were made to accommodate the varying seasonal availability of agricultural by-products between winter and summer. Because the two supplementation periods did not occur simultaneously under identical climatic conditions, comparisons between SH and CF are interpreted as observational associations rather than causal effects of supplement type.

### 2.3. Animal Data Collection

The AMS used on the farm was a commercial single-box robot (Astronaut A5; Lely, Maassluis, The Netherlands), which was installed on 12 January 2020. Data on MY (kg/day) and MF (milking/day) were automatically recorded by the AMS software under routine operations. All records were obtained from cows in their first to third lactation. According to the farm’s management practices, the herd consisted primarily of Holstein cows, with one or two Jersey cows occasionally using the AMS.

To evaluate short-term variability, “change between two consecutive days” (Δ) was defined as:

ΔX_day0_ = X_day1_ − X_day0_,

where X represents MY, MF, THI, dry-bulb temperature, or RH. Thus, Δ reflects changes from the previous day to the current day rather than an arbitrarily selected “2-day period.”

### 2.4. Environmental Data Collection

The ambient temperature (as dry bulb temperature, T_db_, °C) and RH (%) were recorded by a HOBO MX2301A Data Logger (Onset Computer Corporation, Bourne, MA, USA). The sensor was placed on the feed bunk railing, which was approximately 4 m from the AMS, at a height of 2.5 m. Data were collected at hourly intervals. The same data logger was used throughout the study to ensure consistency. The sensor was positioned away from the wind direction to minimize interference from water droplets produced by the mist and sprinkler systems. To ensure data accuracy, the data logger was regularly cleaned to prevent dust accumulation.

Daily mean T_db_ and RH were calculated from the hourly records. The THI used in this study, adopted by the National Research Council [18] based on the original formulation by Thom [19], was calculated using the following equation:

THI = (1.8 × T_db_ + 32) − (0.55 − 0.0055 × RH) × (1.8 × T_db_ − 26.8)

To evaluate lagged effects, THI values were also summarized for the same day (day 0) and for prior days (day −1, day −2, day −3). For example, THI on day −3 is the mean THI recorded three days before the MY/MF outcome day.

### 2.5. Data Management and Statistical Analysis

Milking and management data were automatically recorded by the AMS during routine husbandry management from 29 March 2021 to 28 March 2023. Because data were collected passively over multiple years, individual-level characteristics (e.g., age, body weight, parity, and breed composition) varied continuously and were not controlled by experimental design. Consequently, breed-specific sensitivity analyses, including exclusion of the occasional Jersey cows, were not feasible in the present dataset. Data were screened to remove records that could bias associations between climate and milking outcomes. Specifically, days were excluded if (1) the AMS malfunctioned (the day of failure and one subsequent day until repair), (2) milking abnormalities persisted for more than 6 h, (3) the AMS cow population increased by >10% compared with routine levels, or (4) the day corresponded to the supplement replacement date and the subsequent three days to account for adaptation and short-term disturbances. After applying these criteria, the final dataset contained 700 valid daily observations. Because the study used passively collected farm records, a priori sample size calculation was not performed. Instead, all available daily observations meeting the predefined quality criteria were included to maximize representativeness under real-world conditions.

The unit of analysis was the daily herd observation. All analyses were conducted using SPSS (version 22; IBM, Armonk, NY, USA), and Akaike information criterion (AIC) [20] comparisons for breakpoint selection were conducted using Microsoft Excel (Microsoft 365). Distributions of MY, MF, T_db_, RH, and THI, as well as model residuals, were evaluated using histograms, Q–Q plots, and Shapiro–Wilk tests. Because normality was not met for multiple variables and/or residuals (*p* < 0.05), non-parametric methods were used where appropriate. Associations among MY, MF, days in milk (DIM), herd size, and environmental variables were therefore evaluated using Spearman rank correlations (denoted as *r*_s_). To avoid multicollinearity among closely related environmental indicators, THI, T_db_, and RH were analyzed in separate models rather than entered simultaneously.

Environmental thresholds for MY and MF were identified using piecewise regression models. To determine the optimal thresholds, candidate breakpoints were systematically tested across the observed ranges of environmental variables at fixed intervals (0.5 °C for T_db_, 5% for RH, and 1 unit for THI). Candidate breakpoints were compared using AIC, and the breakpoint associated with the lowest AIC was retained as the threshold [10]. Uncertainty around the estimated breakpoints was evaluated using the AIC difference (∆AIC), defined as ∆*AIC* = *AIC_i_* − *AIC_min_*. Candidate breakpoints with a ∆*AIC* < 2 were considered to have substantial statistical support, thereby defining the confidence set of the threshold. In cases where multiple local minima in AIC were observed across the temperature range, the first (lowest temperature) statistically supported breakpoint was selected as the critical threshold. Furthermore, this selection aligns with the visual patterns of the breakpoint graphs, which reflect the physiological manifestation of the cows transitioning from an initial stable state to an unstable phase. From a biological and herd management perspective, identifying the initial onset of heat stress (the first inflection point) provides earlier and more actionable warning for preventive cooling interventions than selecting a secondary, higher-temperature breakpoint.

The threshold was defined as the corresponding x-axis value at the selected breakpoint, and slopes (denoted as the b-value) below and above the threshold were estimated separately. Additionally, simple linear regressions were performed on the entire dataset to estimate the overall linear effects (overall slopes) of the climatic variables. For the soybean hull (SH) versus concentrate feed (CF) comparison, unadjusted period means are presented descriptively only. Because the dataset consisted of repeated daily observations collected sequentially from the same herd, no independent-samples t-test was used. Instead, exploratory adjusted comparisons between periods were performed using separate analyses of covariance (ANCOVA), with supplementation period as a fixed factor and THI, DIM, or herd size entered individually as covariates. Results are reported as estimated marginal means ± SE with 95% confidence intervals. Statistical significance was set at *p* < 0.05; exact *p* values are reported where applicable, except when *p* < 0.001.

## 3. Results

### 3.1. Environmental Data

All data collected during times when the AMS malfunctioned, including the day of the AMS failure and the subsequent days until repair, were excluded from our analysis. In addition, we excluded data corresponding to days when milking abnormalities lasted >6 h and those corresponding to periods when the AMS cow population increased by >10%. Data from the day of feed replacement and the subsequent three days were excluded from the analysis to account for the cows’ adaptation period and to avoid bias from short-term disturbances in feeding and milking behavior. Finally, 700 valid data were obtained, including 339 and 361 valid entries pertaining to the SH and CF periods, respectively. Over the 2-year study period, the average day in milk (DIM), herd size, MY, and MF were 207.6 ± 36.0 days, 20.0 ± 2.2 cows, 26.7 ± 3.4 kg/day, and 2.39 ± 0.26 times/day, respectively.

**Figure 2:**
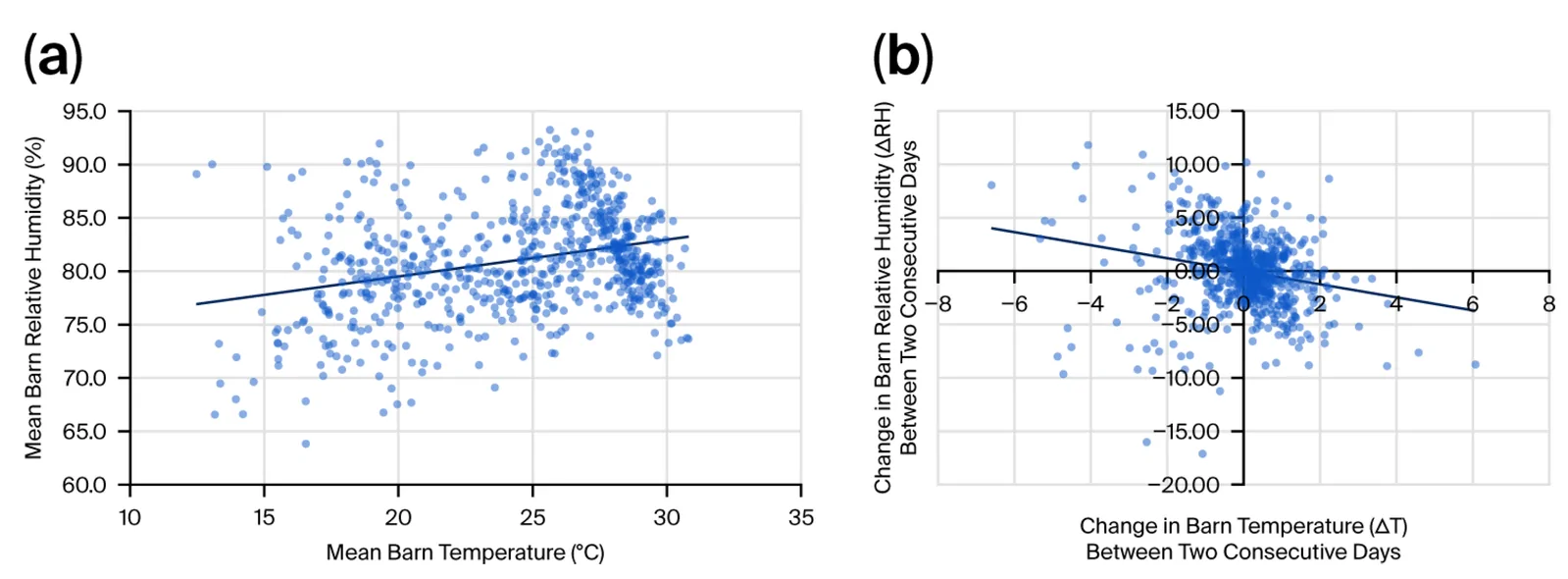
In-barn conditions and short-term climatic variations during the experimental period. (**a**) Relationship between daily mean barn temperature and mean relative humidity throughout the experimental period, showing an overall pattern of higher relative humidity at higher barn temperatures. (**b**) Correlation between day-to-day changes in barn temperature (ΔT) and relative humidity (ΔRH) between two consecutive days, indicating that short-term decreases in barn temperature were associated with increases in relative humidity.

Between 29 March 2021 and 28 March 2023, the barn’s average T_db_, RH, and THI were 24.3 °C ± 4.2 °C (daily averages ranging from 12.5 to 30.8 °C), 81.1% ± 5.0%, and 73.8 ± 6.9 (Table 2). Seasonal variation was observed in the barn environment, as per the local climatic patterns in Taiwan (spring: March–May; summer: June–August; fall: September–November; and winter: December–February). During summer, the barn’s average T_db_, RH, and THI were 28.1 °C ± 1.2 °C (daily averages ranging from 24.7 to 30.7 °C), 84.1% ± 4.0%, and 80.3 ± 1.5, respectively, correlating with MF and MY of 2.25 ± 0.17 times/day and 24.9 ± 2.5 kg/day. By contrast, during winter, the average T_db_, RH, and THI were 18.7 ± 2.2 °C, 79.2% ± 4.9%, and 64.6 ± 3.5, respectively, correlating with MF and MY of 2.56 ± 0.22 times/day and 27.7 ± 3.8 kg/day. A significant positive correlation was discovered between T_db_ and RH (*p <* 0.001; a slope of 0.345; Spearman’s rank correlation r = 0.232; Figure 2a). Regarding changes in environmental factors over a period of 2 consecutive days, changes in RH (∆RH) exhibited a significant negative correlation with changes in T_db_ (∆T_db_; Figure 2b). The results showed that the overall climate pattern in the study area indicated a positive correlation between T_db_ and RH, whereas short-term weather fluctuations were characterized by increases in RH accompanied by reductions in T_db_.

### 3.2. Correlations Between Herd Size, MF, and MY

**Figure 3:**
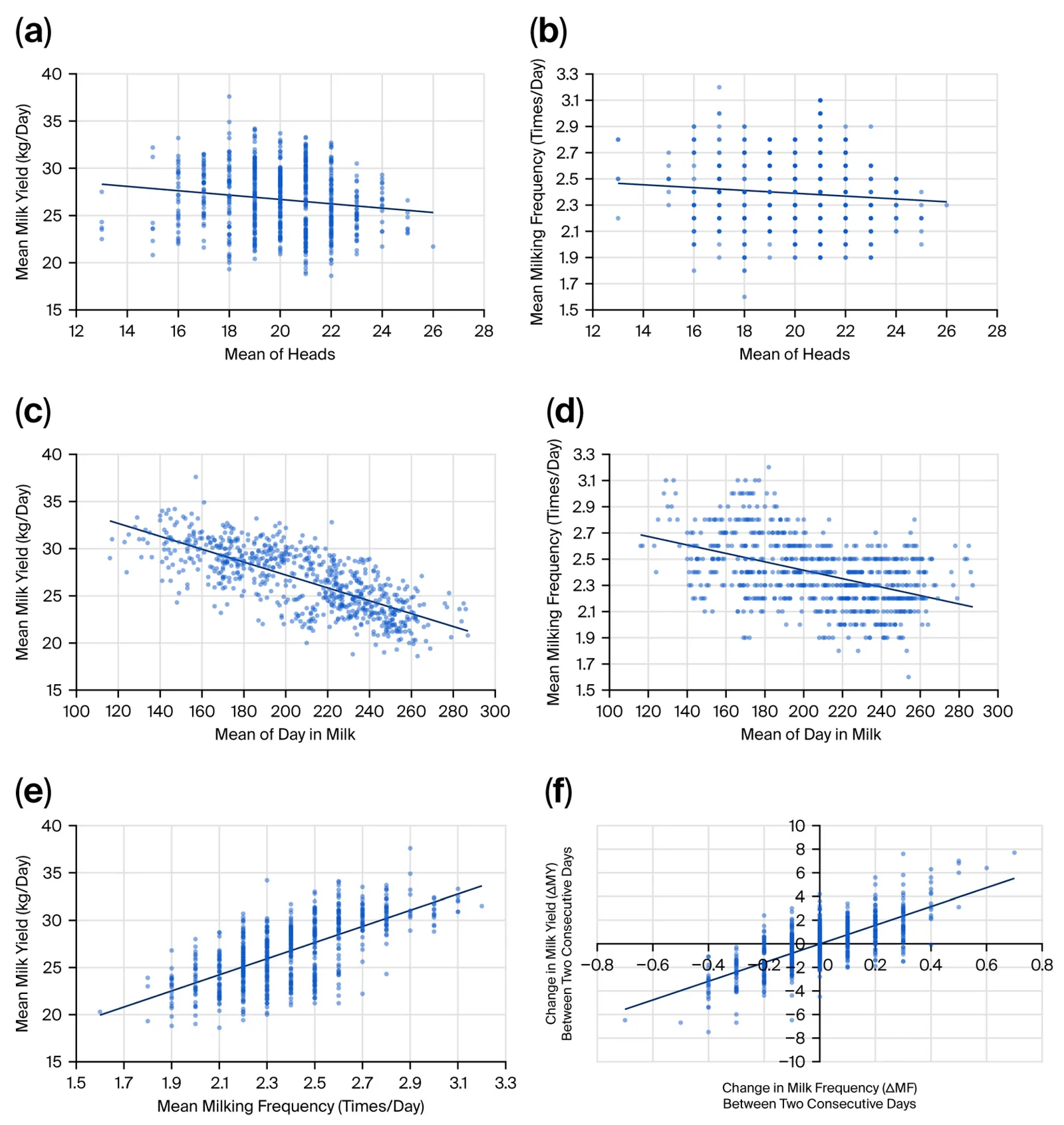
Correlations between herd size, DIM, MF, and MY. Each hollow circle indicates a specific value, with darker shades representing more frequent occurrences of that value. MY and MF were significantly and negatively correlated (*p <* 0.001 and 0.024 respectively) with herd size (**a**,**b**) and DIM (**c**,**d**). MF was significantly and positively correlated (*p <* 0.001) with MY (**e**). Changes in MF were significantly and positively correlated (*p <* 0.001) with changes in MY (**f**). MF, milking frequency; MY, milk yield.

Both MY (*p <* 0.001) and MF (*p =* 0.024) slightly decreased with increasing herd size (MY r_s_ = −0.167; MF r_s_ = −0.086; Figure 3a,b; Table 3). In addition, DIM was significantly negatively correlated with both MY and MF (MY r_s_ = −0.741; MF r_s_ = −0.432; *p <* 0.001; Figure 3c,d). The results indicated that for every 100-day increase in DIM, MY decreased by 6.8 L and MF decreased by 0.3 times/day. A significant positive correlation was observed between MF and MY (*p <* 0.001; r_s_ = 0.644; Figure 3e), with a slope of 8.547, indicating that each additional milking session was associated with an increase of 8.547 L in MY. Similarly, changes in MF (∆MF) and MY (∆MY) over a two-day period were also significantly positively correlated (*p <* 0.001; r_s_ = 0.686; Figure 3f; Table 4). With respect to thresholds, no significant breakpoint could be determined for the effect of MF on MY, or for the effect of DIM on MY and MF.

### 3.3. Effects of Various Environmental Factors on MY and MF

MY and MF were significantly negatively correlated with THI, T_db_, and RH (Table 3). For each unit increase in THI, MY decreased by 0.179 kg/day (r_s_ = −0.408), and MF decreased by 0.021 times/day (r_s_ = −0.615). For every 1 °C increase in T_db_, MY decreased by 0.290 kg/day and MF decreased by 0.034 times/day. For every 10% increase in RH, MY decreased by 0.752 kg/day and MF decreased by 0.1 times/day. Changes in ∆THI, ∆T_db_, and ∆RH over a two-day period had no significant effects on ∆MF or ∆MY.

**Figure 4:**
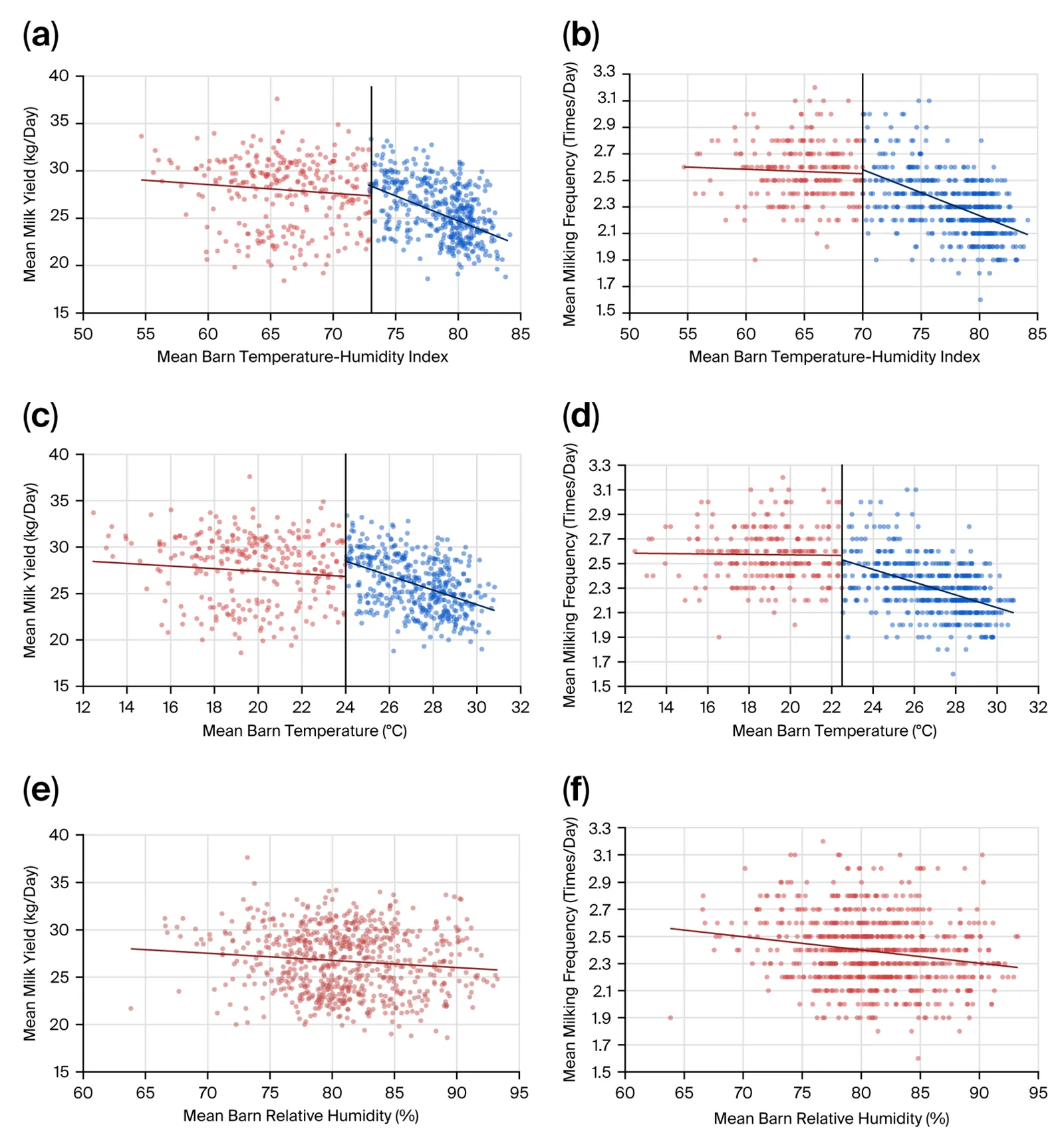
Effects of THI, temperature, and RH on MY and MF. Each hollow circle represents a data point, with darker shades indicating higher frequencies of occurrence. The optimal model was identified using piecewise regression and the Akaike information criterion. The x-axis breakpoints correspond to the THI thresholds beyond which MY and MF declined sharply at various rates. The THI thresholds identified for MY and MF were 73 (**a**) and 70 (**b**), respectively. The temperature thresholds for MY and MF were 24.0 °C (**c**) and 22.5 °C (**d**), respectively. Although no distinct threshold was observed for RH (**e**,**f**), it was significantly and negatively correlated with MY and MF. MF, milking frequency; MY, milk yield; RH, relative humidity; THI, temperature–humidity index.

The THI and T_db_ thresholds for MY were 73 and 24.0 °C, respectively (Figure 4a,c). Analysis of candidate breakpoints confirmed the high accuracy of these estimates. Specifically, the threshold at THI 73 was robustly supported with minimal statistical uncertainty, as no other candidate values fell within the ∆AIC < 2 confidence range. Below these thresholds, MY was not significantly correlated with either THI or T_db_ (*p* > 0.05); however, beyond the thresholds, MY declined significantly (*p <* 0.001; Table 3). The corresponding slopes were −0.522 and −0.782, indicating that for each unit increase in THI, MY decreased by 0.522 kg/day, and for every 1 °C increase in T_db_, MY decreased by 0.782 kg/day.

The THI and T_db_ thresholds for MF were 70 and 22.5 °C, respectively (Figure 4b,d). While evaluation of the surrounding candidate breakpoints revealed a broader confidence set ranging from THI 69 to 72 and 21.0 to 24.0 °C (∆AIC < 2) for MF—reflecting a potentially more gradual behavioral transition under heat stress—these selected thresholds provided the optimal mathematical fit (statistically supported local minimum AIC) to accurately identify the initial onset of the decline. Below these thresholds, MF did not change significantly with increases in T_db_ (*p* = 0.511) and showed only a slight decline with THI (*b* = −0.003 times/day per unit, *p* = 0.014; Table 3). Beyond the thresholds, MF declined significantly, decreasing by 0.035 times/day for each unit increase in THI and by 0.051 times/day for each 1 °C increase in T_db_ (both *p <* 0.001). Notably, no significant thresholds were identified for the effects of RH on either MY or MF (Figure 4e,f).

MY and MF were significantly negatively correlated with daily THI values on days 0, −1, −2, and −3 (*p <* 0.001; Table 5). Among these, the correlations for MY showed a slight progressive increase up to day −3 (r_s_ ranging from −0.408 to −0.445), while the correlations for MF remained consistently stable across the lag periods (r_s_ ranging from −0.614 to −0.617).

### 3.4. Comparison of MY and MF Between the SH and CF Periods

In the adjusted analyses, MY was higher in the CF period than in the SH period when THI, DIM, or herd size were controlled separately (Table 6). Under the same THI, estimated marginal MY was 27.56 ± 0.16 kg/day in the CF period and 25.78 ± 0.16 kg/day in the SH period (*p* < 0.001). Similarly, MF was higher in the CF period after adjustment for THI (2.41 ± 0.01 vs. 2.37 ± 0.01 times/day, *p* = 0.004) and herd size (2.42 ± 0.01 vs. 2.36 ± 0.01 times/day, *p* = 0.009), whereas no significant difference was detected after adjustment for DIM.

**Figure 5:**
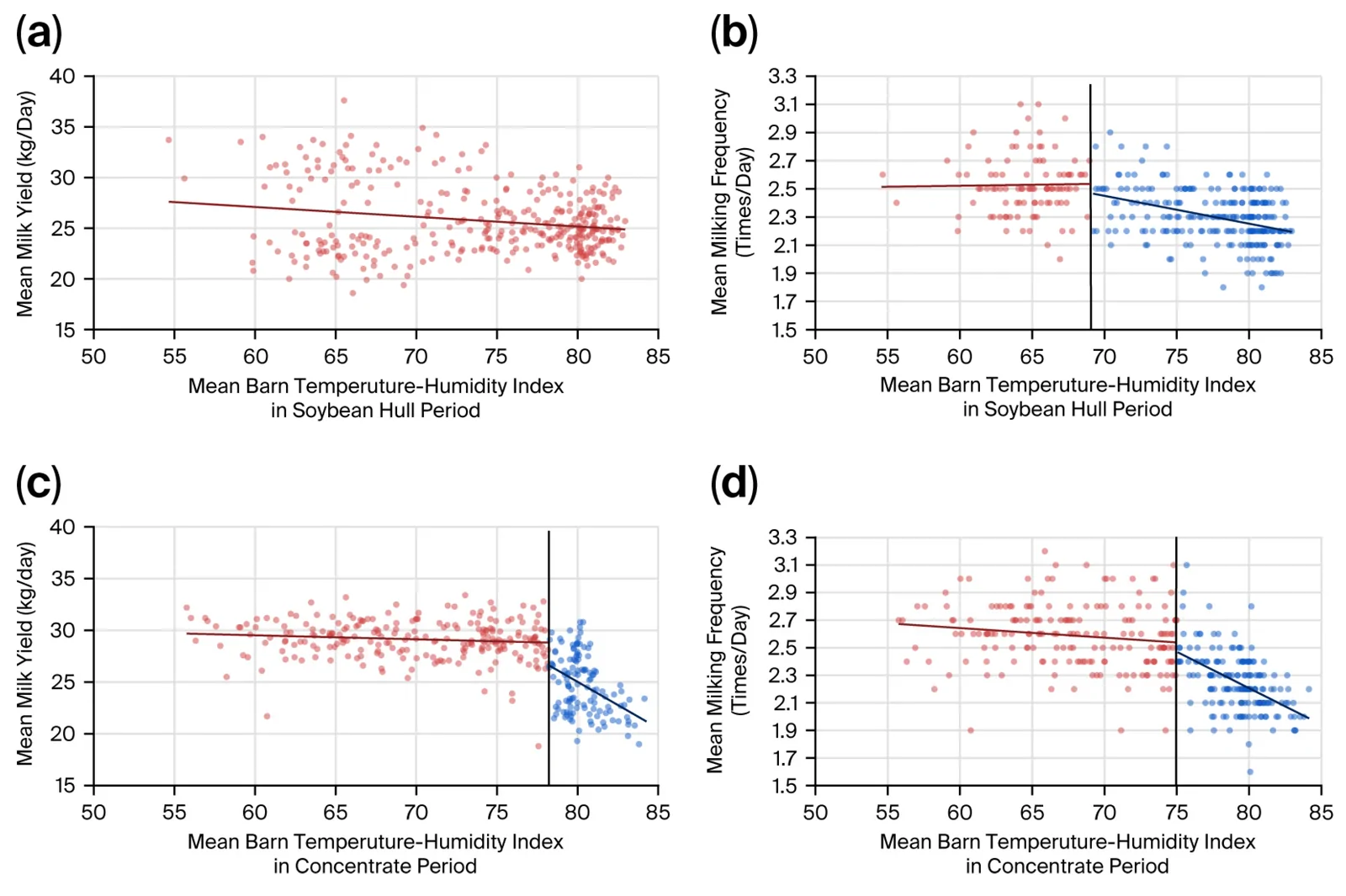
Effects of THI on MY and MF during the soybean hull (SH) period and concentrate feed (CF) period. Each hollow circle denotes a value, with darker shades indicating higher frequencies of occurrence. The optimal model was identified through piecewise regression and Akaike information criteria. The breakpoints on the x-axis represent the THI thresholds, beyond which marked declines were noted in MY and MF. In the soybean hull period, the THI thresholds for MY and MF were unobserved (**a**) and 69 (**b**), respectively. In the concentrate feed period, the THI thresholds for MY and MF were 78 (**c**) and 75 (**d**), respectively. MF, milking frequency; MY, milk yield; THI, temperature–humidity index.

During the SH period, no significant THI threshold for MY was detected. In contrast, the THI threshold for MY during the CF period was identified as 78. Below this threshold, THI exerted no significant effect on MY; however, beyond the threshold, each unit increase in THI resulted in a reduction of 0.881 kg/day in MY (Figure 5a,c).

Regarding MF, the THI thresholds were 69 and 75 during the SH and CF periods, respectively (Figure 5b,d), which differed from the overall threshold (70). In the SH period, MF showed no significant decline below the threshold (*p* = 0.646), whereas above the threshold, MF decreased by 0.02 times/day for each unit increase in THI (Table 7). In contrast, during the CF period, MF was sensitive to THI regardless of whether the value was below or above the threshold: below the threshold, MF decreased by 0.007 times/day for each unit increase in THI (*p* = 0.010), whereas above the threshold, the decline reached 0.053 times/day (*p <* 0.001).

## 4. Discussion

### 4.1. The Effects of Herd Size and DIM on MF and MY

Castro et al. [21] investigated 29 U.S. dairy farms using AMSs and found that increasing herd size resulted in reduced MF. Similarly, King et al. [22] demonstrated a negative correlation between MF, herd size, and the cow–AMS ratio. Under normal operating conditions, cow queuing to enter the AMS can be routinely observed [23]. Increasing both the maximum allowed visits per cow (five in this study) and the number of cows raises the overall AMS service demand, creating long queues. As a result, cows less willing to wait reduce their visits, thereby lowering the average milking frequency [24]. These findings explain the present study’s observation that MF decreased by 0.011 visits for each additional cow per AMS.

Milking frequency exerts a significant influence on MY. In conventional milking systems, shifting from two to three daily milkings can increase milk production by approximately 3.5 kg/day [25]. The implementation of AMSs increased the average milking frequency per cow, thereby enhancing MY [26]. A meta-analysis of 43 studies conducted under pasture-based systems reported that each additional milking visit per day was associated with a 14.1 kg/day increase in MY [27]. Similarly, an analysis of 44 AMS farms indicated that average milk yield increased by approximately 3% following AMS adoption, with improvements reaching up to 9% after more than two years of use [28]. Another study also documented increases of up to 12% [1]. These results support the present study, which identified a significant positive correlation between MF and MY (*p <* 0.001; r_s_ = 0.644), with MY increasing by 8.547 kg/day for each additional milking per day.

Determinants of MY in AMSs are not restricted to MF but also include factors such as diet composition and feeding schedules [1], parity [29], milking failure rates [29], and DIM. The present results showed that an increase of 100 DIM was associated with a significant decline of 0.3 milkings/day. This finding is consistent with Lessire et al. [27], who reported that early-lactation cows displayed MF values higher by 0.34 milkings/day compared with late-lactation cows.

### 4.2. Effects of T_db_ on MY and MF

The effects of temperature and heat stress on dairy cows are often discussed together. The upper limit of the thermal comfort zone for dairy cows is reported to be 25 °C to 26 °C [30,31]. When ambient temperature exceeds this range, cows experience heat stress, which alters behavioral patterns, including increased aggressive behavior [32] and reduced lying time [33]. Heat stress also decreases dry matter intake (DMI), consequently reducing MY [34]. The results of this study also confirmed these findings, showing that, overall, for each 1 °C increase in T_db_, MF and MY decreased by 0.034 times/day and 0.290 kg/day, respectively.

However, the relationship between environmental factors and animal physiological responses is not necessarily linear [12]. In many cases, segmented or breakpoint regression provides greater explanatory power than simple linear analysis [35], because animals may show distinct response patterns once environmental stress surpasses a certain threshold. When environmental indices exceed such thresholds, animal behavior and physiological responses often shift into different patterns [10,36]. In the present study, piecewise regression was applied, and the AIC was used to determine the optimal model. AIC was selected because it enables objective comparison between linear and piecewise regression models, which is crucial given the potential non-linear responses of cows to heat stress.

The thresholds identified in the present study were 22.5 °C for MF and 24.0 °C for MY. Below these thresholds, T_db_ had no significant effect on MF or MY; however, once exceeded, MF and MY declined sharply, at rates of −0.051 times/°C and −0.782 kg/°C, respectively. Notably, both T_db_ thresholds were lower than the traditionally reported upper limit of the thermal comfort zone. Similar findings have been reported by Zhou et al. [37], who observed changes in rectal temperature and respiratory rate between 19 °C and 26 °C, and by Li et al. [12], who found that rectal temperature began to increase once the ambient temperature exceeded 20.4 °C. These results suggest that heat stress responses may occur even within the traditionally defined comfort zone. Overall, our findings highlight that, within AMSs, the negative effects of heat stress on MF and MY can manifest at relatively moderate ambient temperatures. This emphasizes the importance of close monitoring of barn conditions and proactive implementation of cooling strategies, even before the upper critical temperature of the thermal comfort zone is reached.

### 4.3. Effects of RH on MY and MF

Relative humidity has a complex relationship with apparent temperature in dairy cows under heat stress [38]. When ambient temperature exceeds 15 °C, higher RH increases the apparent temperature experienced by cows, thereby aggravating heat stress. Conversely, at lower temperatures, higher RH may have a moderating effect [38]. In dry-heat environments, RH has a positive impact on rectal temperature and respiratory rate, showing a negative association with heat stress responses [5]. In contrast, under humid conditions, RH adversely affects respiratory rate, rectal temperature, and lactational performance. For instance, Zhou et al. [37] demonstrated that with increasing temperature, the decline in feed intake was more pronounced under high-humidity conditions (60–90%) than under moderate (45–70%) or low (30–50%) humidity. Another study reported that even at relatively low ambient temperatures, prolonged exposure to high RH (>90%) elevated rectal temperature, restricted feed intake, and reduced MY [39,40].

The results of this study revealed a weak but significant negative correlation between RH and both MF and MY. This finding is consistent with previous reports that elevated RH intensifies heat stress responses in dairy cattle [39,40]. This contrasts with the results of Dikmen and Hansen [5], who presented that heat stress decreased with increasing RH. This outcome may be explained by the climatic characteristics of the present study site, where higher temperatures are typically accompanied by higher humidity. This combination increases heat stress by inhibiting cutaneous evaporation and impairing effective heat dissipation through sweating [41]. Although short-term weather changes (e.g., ∆T_db_ and ∆RH) may be driven by rainfall or cloud cover, which reduce temperature while increasing humidity, such variations did not significantly affect short-term changes in MF and MY (Table 4).

With respect to thresholds, no critical RH value was identified, unlike the T_db_ threshold. From a management perspective, the absence of a clear RH threshold does not diminish its importance. High humidity still suppresses evaporative heat loss, thereby amplifying the negative effects of elevated temperature. This presents a dilemma for dairy farmers, as common cooling strategies such as sprinklers and misters effectively lower ambient temperature but simultaneously increase RH. Therefore, careful calibration of cooling systems is essential to balance their benefits and drawbacks.

### 4.4. Effects of THI on MY and MF

The THI calculation formula proposed by the Council [18] remains one of the most widely used indices for evaluating heat stress in cattle. THI has been shown to influence MF in AMSs [42]. Morales-Piñeyrúa et al. [43] reported that each unit increase in THI reduced voluntary MF by 0.002 times/day, while Bouraoui et al. [13] observed, in conventional milking systems, that MY declined by 0.41 kg/day once THI exceeded 69. Similarly, under AMS conditions, Morales-Piñeyrúa et al. [43] found that each unit increase in THI reduced MY by 0.40 kg/day. In contrast, the present study revealed larger effects, with each unit increase in THI associated with overall decreases of 0.021 times/day in MF and 0.179 kg/day in MY. However, this overall linear effect masks the true non-linear dynamic of heat stress.

Thresholds serve as important management references. Armstrong [44] defined THI < 72 as comfortable and 72 ≤ THI < 78 as mild heat stress. Thus, THI 72 can be defined as a threshold value. The results of the present study showed that the THI thresholds for MF and MY were 70 and 73, respectively, values close to Armstrong [44] definition and to those reported by Gálik et al. [45]. Below these critical thresholds, the herd demonstrated an initial resilience, MF and MY showed little variation with THI. Conversely, once the threshold was exceeded, a marked reduction occurred, with each unit increase in THI reducing MF and MY by 0.035 times/day and 0.522 kg/day, respectively. These results are consistent with Wildridge et al. [11], who found that when THI was below the threshold, MF decreased only slightly (0.005 times/day per unit), but declined sharply when THI exceeded 75 (0.045 times/day per unit).

Notably, the MF threshold in the present study (70) was lower than that reported by Wildridge et al. [11] (75), but both were considerably higher than the thresholds for lying behavior, which ranged between 67 and 68 [10,33]. These results should be interpreted with caution, as MF in AMSs is influenced not solely by heat stress but also by herd size, feed incentives and management practices such as guiding cows into the AMS. Without such human intervention, actual MF could be lower and the threshold subject to change. Similarly, the MY results should be applied with caution.

It is noteworthy that the THI thresholds were almost identical to those for T_db_, as at an average RH of 80%, the corresponding T_db_ values were 24.0 °C and 22.5 °C. This similarity arises not only because humidity does not exhibit an obvious threshold, but also because in commonly used composite indices, temperature carries a much greater weight than humidity, making its variation far more influential [46]. For example, at RH = 80%, each 1 °C increase in temperature raises THI by 1.6 units, whereas at 24 °C, a 10% increase in RH raises THI by only 0.9 units. Previous studies have also indicated that the correlation coefficient of T_db_ with physiological indicators is only slightly lower than that of THI [5].

Although composite environmental indices such as THI are often recommended for evaluating heat stress, they may not fully capture the effects of humidity in tropical and subtropical regions; furthermore, varying the weightings of temperature and humidity across different THI formulas can lead to inconsistent threshold values [47]. While THI models applied in this study produced results highly consistent with T_db_, models that assign greater weight to humidity have been shown to provide better explanatory power under hot-humid conditions [46]. Therefore, the development of humidity-sensitive indices specifically tailored to these regions could improve threshold estimation accuracy and provide more reliable guidance for herd management decisions.

### 4.5. Weather Change and Its Delayed Effect on MY and MF

Several studies have demonstrated that weather variations can exert delayed effects on production performance in dairy cows. Morales-Piñeyrúa et al. [43] reported that MY was more strongly affected by THI three days prior than by the current day. Similarly, an evaluation using AIC in AMSs revealed that the best model fit occurred when THI values from previous days were included [46]. Consistent with these findings, the present study revealed that MY was significantly linearly correlated with THI from day 0 to day −3 (*p <* 0.001; Table 5), with the correlation increasing progressively from day 0 to day −3. Although the correlation coefficients across the evaluated lag periods were relatively close, this gradual pattern suggests that the negative impact of heat stress on milk yield may accumulate over time, rather than being limited to immediate weather conditions. Farm managers must not passively wait until the THI exceeds the threshold on the current day; instead, they should integrate weather forecasts to anticipate heat load several days in advance and initiate proactive, preventive cooling interventions.

One plausible explanation is that increases in THI reduce dry matter intake, which in turn influences milk yield [7]. If cows are unable to dissipate body heat effectively during the night, the physiological burden may persist into the following day, thereby intensifying the negative effects on production [36]. Unlike short-term physiological responses such as respiratory rate, which can recover relatively quickly, reductions in DMI tend to persist under prolonged heat stress exposure [48], thereby explaining the stronger lagged association observed for MY.

Interestingly, this delayed effect was not as evident for MF in the present study. Correlations between MF and THI remained similar across day 0 to day −3 (ranging from −0.614 to −0.617), suggesting that MF is influenced not only by climatic stress but also by management interventions, such as guided strategies or feed supplementation, that may mask seasonal lag patterns.

It is also important to consider whether the observed cumulative trend applies across all seasons or is primarily driven by summer conditions. Seasonal differences in baseline temperature and humidity may influence the degree of heat load accumulation. During prolonged hot and humid periods, such as summer in subtropical regions, the inability to dissipate heat overnight may lead to stronger carry-over effects on MY. In contrast, in cooler seasons cows may recover more quickly from transient heat stress, resulting in weaker lagged associations. Future research stratified by season will be essential to clarify whether the lagged association is a universal phenomenon or season-specific.

### 4.6. Differences of MY and MF Between the SH and CF Periods

In AMSs, supplementary feed serves not only as a source of nutrition but also as the primary incentive for cows to voluntarily enter the AMS [49]. The attractiveness and composition of supplementary feed strongly influence both MF and MY [1]. Farms that provide supplementary feed are able to maintain higher MF and MY [27].

During routine farm management, two supplementary feeding periods were observed: an SH period followed by a CF period. After adjustment for THI and herd size, both MF and MY were higher in the CF period than in the SH period (Table 6). For example, under the same adjusted THI, MF averaged 2.41 ± 0.01 times/day in the CF period and 2.37 ± 0.01 times/day in the SH period, whereas MY averaged 27.56 ± 0.16 and 25.78 ± 0.16 kg/day, respectively. The observed THI thresholds also differed between periods. The MF threshold was 69 in the SH period and 75 in the CF period, whereas an MY threshold was identified only in the CF period (78).

Previous studies have suggested that composition or palatability of AMS concentrate can influence voluntary AMS visits [50], whereas other studies have reported limited effects [51]. The present observational results indicate that the CF period was associated with higher MF, MY, and altered THI thresholds. However, because these two feeding periods occurred sequentially, the supplement type is inherently confounded with ongoing herd management changes. While these findings suggest that feed characteristics might influence cows’ motivation to voluntarily visit the AMS, these period-specific differences cannot be causally attributed to the feed change alone.

In terms of magnitude, MF changed little below the observed threshold but declined more steeply once the threshold was exceeded, particularly in the CF period. Above the observed threshold, MF declined by 0.053 visits per THI unit in the CF period compared with 0.020 visits in the SH period. These results indicate that even when the observed thresholds differed between periods, heat stress remained strongly associated with reduced AMS performance once threshold conditions were exceeded.

Taken together, the CF period showed higher adjusted average MF and MY and higher observed THI thresholds than the SH period. From a herd-management perspective, the main practical implication is that MF and MY remained vulnerable once THI exceeded the observed thresholds, reinforcing the importance of cooling and threshold-based monitoring in hot-humid AMS conditions.

It should be emphasized that cows’ willingness to enter the AMS is influenced not only by the incentive of supplementary feed but also by herd management, facility design, and animal-related factors [52]. With changes in farm configurations (for example, a transition from a free-flow to a guided-flow system) the thresholds for MY and MF may also be altered. Therefore, threshold values should not be regarded as fixed; instead, they should be regularly monitored and used to inform management adjustments. In the present study, although the CF period was associated with higher observed THI thresholds for MY and MF, these findings should be interpreted cautiously as management references rather than direct evidence of a causal feed effect. Further interventional studies comparing CF, SHs, or alternative supplementary feeds are warranted to identify the most suitable strategies for optimizing milk production efficiency and economic returns in high-temperature, high-humidity environments.

Several limitations should be considered when interpreting these findings. First, this was a single-farm study, and the analyses were based on daily herd-level observations collected retrospectively under routine management. Because these daily measurements were taken from the same herd, they are not strictly independent experimental units. Furthermore, due to the dynamic changes in herd composition (e.g., cows drying off and new heifers entering) during the study period, we analyzed aggregated daily averages and did not explicitly account for temporal autocorrelation in our statistical models. Consequently, the results may not be directly generalizable to farms with different AMS configurations, herd structures, climates, or management practices. Second, because the dataset was passively collected, potentially important animal- and nutrition-related variables, including herd composition, age, body weight, parity, breed composition, individual dry matter intake, and complete diet composition, were not standardized or fully available for analysis. Third, the SH and CF periods occurred sequentially rather than concurrently and were therefore exposed to different climatic histories; consequently, differences between periods cannot be attributed solely to supplement type. Moreover, given that the supplementary feed volume was directly tied to daily production, the observed yield variations between the two periods might reflect a mutual influence between supplement dosage and milk yield, rather than the supplement composition alone. Finally, although the herd consisted primarily of Holstein cows, one or two Jersey cows were occasionally present, and breed-specific sensitivity analyses were not feasible within the present daily herd-level framework. Accordingly, the threshold values reported here should be strictly interpreted as farm-specific management references rather than universally fixed biological thresholds. Future multi-farm and interventional studies with concurrent feeding comparisons and more complete cow-level phenotype and intake data are needed to confirm and refine these thresholds.

### 4.7. Recommendations for Using AMSs in Hot and Humid Environments

Automatic milking systems are regarded as a solution to labor shortages in dairy farms. Cows’ willingness to voluntarily visit the AMS is a key factor influencing its utilization; if cows do not actively visit the machine, alternative strategies are required to increase milking frequency. Guided-flow systems can effectively improve daily milking frequency [29]. However, guided-flow may lead to prolonged waiting times, which can negatively impact cow welfare. In particular, low-ranking cows may spend up to 15 times longer waiting compared with high-ranking cows [24]. Although manual guiding can effectively increase MF, this practice may affect the stockperson–cow relationship [53], further compromising animal welfare. In addition, manual guiding may increase labor costs, which contradicts the original goal of introducing AMSs to reduce labor requirements [54].

However, climatic conditions, particularly heat stress, significantly influence cows’ willingness to visit the AMS [52]. Speroni et al. [2] reported that hot seasons reduced milk production in both CMSs and AMSs, but the reduction was more pronounced in AMSs (−3.0 ± 0.8 vs. −4.5 ± 0.6 kg/day). Cows milked in AMSs, especially high-yielding and multiparous cows, were found to be more susceptible to heat stress [55]. Although AMSs can increase both MF and MY, the management of heat stress remains a major challenge [52].

Based on farm management data from this study, the observed MF threshold was 69 during the SH period and 75 during the CF period. In practice, a large proportion of days still exceeded these thresholds. According to the number of data collection days, mean THI ≥ 69 and 75 accounted for as much as 71.64% and 51.64% respectively during the two years (Table 8), which is equivalent to nearly half a year requiring increased guiding intensity. This indicates that increased guiding intensity, cooling, or other herd-management adjustments may be needed during hot periods.

In the future, according to IPCC projections, the global average temperature may increase by 1.5 °C between 2030 and 2052, with mid- and high-latitude regions experiencing rises of up to 3 °C and 4.5 °C, respectively [56]. In Taiwan, climate change has already altered seasonal patterns, and future projections suggest that summer may extend to 155–210 days [57]. These projections highlight that the challenges posed by heat stress on dairy production will become increasingly severe. It should be noted that THI plays a critical role in explaining behavioral variations within a certain range [58], but the impacts of extreme heat events may be more severe than anticipated. The dual goals of AMSs—reducing labor and improving animal welfare—may be more difficult to achieve in tropical and subtropical regions compared with temperate areas. Future research evaluating the performance of AMSs in hot and humid regions and developing appropriate strategies to encourage cows to voluntarily visit AMSs is recommended.

## 5. Conclusions

This study aimed to evaluate how climatic factors and a management-driven feed change influenced milking frequency and milk yield in an automatic milking system (AMS) under hot-humid conditions, specifically identifying critical environmental thresholds. In this AMS herd, milking frequency and milk yield declined when THI exceeded approximately 70–73 and when T_db_ exceeded 22.5–24.0 °C. Reflecting a gradual accumulation of heat stress, the negative association between milk yield and THI increased progressively. In the observational comparison of sequential feeding periods, the concentrate feed period was associated with higher adjusted milking frequency and milk yield than the soybean hull period; however, because the two supplementation periods were sequential and non-concurrent, these differences should not be interpreted as causal effects of supplement type. Given the single-herd observational design, these thresholds represent farm-specific management references rather than universal biological limits. Ultimately, farm-specific threshold monitoring and timely cooling interventions are highly recommended to support AMS management in hot-humid environments.

## Figures and Tables

**Table 1:** Nutrient composition of soybean hulls and concentrate feed.

	Soybean Hulls (~25 March 2022)	Concentrate Feed (After 25 March 2022)
Crude fat (%)	3.5	3.2
Crude protein (%)	13.7	24.2
Neutral Detergent Fiber (%)	61.6	9.7
Acid Detergent Fiber (%)	43.1	4.9

**Table 2:** In-barn conditions and herd characteristics.

	Barn Temperature (°C) ^1^ (Min–Max)	Barn Relative Humidity (%) (Min–Max)	Barn Temperature–Humidity Index ^2^ (Min–Max)	Herd Size (Cows) ^3^	Days in Milk	Milking Frequency (Times/Day)	Milk Yield (kg/Day)
29 March 2021 to 28 March 2023	24.3 ± 4.2 (12.5–30.8)	81.1 ± 5.0 (63.8–93.3)	73.8 ± 6.9 (54.7–84.1)	20.0 ± 2.2	207.6 ± 36.0	2.39 ± 0.26	26.7 ± 3.4
Spring	24.5 ± 3.3 (15.5–30.8)	78.8 ± 5.0 (66.8–92.0)	73.7 ± 5.2 (60.0–82.9)	21.1 ± 1.6	202.5 ± 33.0	2.46 ± 0.30	28.6 ± 2.8
Summer	28.1 ± 1.2 (24.7–30.7)	84.1 ± 4.0 (77.4–93.3)	80.3 ± 1.5 (75.6–84.1)	20.1 ± 1.9	222.2 ± 30.4	2.25 ± 0.17	24.9 ± 2.5
Autumn	25.8 ± 2.3 (18.9–29.7)	81.9 ± 3.8 (67.7–91.6)	76.3 ± 3.7 (64.8–82.2)	19.8 ± 2.1	203.9 ± 23.9	2.30 ± 0.20	25.8 ± 2.8
Winter	18.7 ± 2.2 (12.5–25.1)	79.2 ± 4.9 (63.8–90.3)	64.6 ± 3.5 (54.7–74.2)	19.2 ± 2.1	199.0 ± 48.1	2.56 ± 0.22	27.7 ± 3.8

^1^ Climatic data (barn temperature, relative humidity, and temperature–humidity index) were calculated based on the continuous 730-day period over the two years, without excluding days with machine malfunctions or other disturbances. ^2^ The temperature–humidity index value was estimated using the equation described by NRC [18]. ^3^ Data for herd size (cows), days in milk, milking frequency (times/day), and milk yield (kg/day) were calculated using only the 700 valid daily observations.

**Table 3:** Results of regression analyses for the effects of DIM, milking frequency, THI, temperature, and RH on daily milk yield and milking frequency.

	Milk Yield				Milking Frequency			
	Data Subset	*r_s_* ^1^	*p*	*b* ^2^	Data Subset	*r_s_*	*p*	*b*
Herd size	All data	−0.167	<0.001	−0.231	All data	−0.086	0.024	−0.011
DIM	All data	−0.741	<0.001	−0.068	All data	−0.432	<0.001	−0.003
MF	All data	0.644	<0.001	8.547	−			
THI	All data	−0.408	<0.001	−0.179	All data	−0.615	<0.001	−0.021
	Below THI threshold (73)		0.077		Below THI threshold (70)	−0.148	0.014	−0.003
	Above THI threshold (73)	−0.453	<0.001	−0.522	Above THI threshold (70)	−0.460	<0.001	−0.035
T_db_	All data	−0.401	<0.001	−0.290	All data	−0.609	<0.001	−0.034
	Below T_db_ threshold (24.0)		0.080		Below T_db_ threshold (22.5)		0.511	
	Above T_db_ threshold (24.0)	−0.434	<0.001	−0.782	Above T_db_ threshold (22.5)	−0.465	<0.001	−0.051
RH	All data	−0.098	0.009	−0.0752	All data	−0.187	<0.001	−0.010

^1^ The *r_s_* value indicates Spearman’s rank correlation. ^2^ The *b* value indicates the slope of the regression equation. DIM is days in milk; MF, milking frequency; THI, temperature–humidity index; T_db_, dry-bulb temperature; RH, relative humidity.

**Table 4:** Results of regression analyses for the effects of changes in THI, temperature, and RH over a 2-day period on milk yield and milking frequency.

	∆Milk Yield ^1^			∆Milking Frequency		
	*r*_s_ ^2^	*p*	*b* ^3^	*r_s_*	*p*	*b*
∆Milking Frequency	0.686	<0.001	7.926	-		
∆THI		0.719			0.264	
∆T_db_		0.595			0.155	
∆RH		0.399			0.141	

^1^ ∆ indicates the changes in values over a period of 2 consecutive days. ^2^ The *r*_s_ value indicates Spearman’s rank correlation. ^3^ The *b* value indicates the slope of the regression equation. THI, temperature–humidity index; T_db_, dry-bulb temperature; RH, relative humidity.

**Table 5:** Spearman correlation coefficients ($r_{s}$) for the associations of lagged THI over a 3-day period with milk yield and milking frequency.

	Day 0		Day-1		Day-2		Day-3	
	*r* *_s_*	*p* ^1^	*r* *_s_*	*p*	*r* *_s_*	*p*	*r* *_s_*	*p*
Milk Yield	−0.408	<0.001	−0.423	<0.001	−0.440	<0.001	−0.445	<0.001
Milking Frequency	−0.615	<0.001	−0.617	<0.001	−0.616	<0.001	−0.614	<0.001

^1^ Significance was set at *p <* 0.05. THI, temperature–humidity index.

**Table 6:** Exploratory comparison of herd characteristics, milk yield, and milking frequency between the soybean hull (SH) and concentrate feed (CF) periods.

	Soybean Hull Period	Concentrate Feed Period	*p* ^1^
Days	339	361	
Herd size	20.3 ± 2.5	19.7 ± 1.6	
DIM	212.3 ± 42.0	202.8 ± 28.6	
THI	74.0 ± 6.9	73.5 ± 6.9	
T_db_	24.5 ± 4.3	24.1 ± 4.2	
RH	80.9 ± 4.9	81.1 ± 5.1	
Milk yield	25.7 ± 3.4	27.6 ± 3.1	
Milking frequency	2.36 ± 0.22	2.42 ± 0.28	
Milk yield—Estimated Marginal Means with same THI (Upper limit–lower limit)	25.78 ± 0.16 (25.46–26.01)	27.56 ± 0.16 (27.25–27.87)	<0.001
Milk yield—Estimated Marginal Means with same DIM (Upper limit–lower limit)	26.06 ± 0.12 (25.83–26.30)	27.30 ± 0.12 (27.07–27.53)	<0.001
Milk yield—Estimated Marginal Means with same heads (Upper limit–lower limit)	25.79 ± 0.18 (25.45–26.14)	27.55 ± 0.17 (27.22–27.89)	<0.001
Milking frequency—Estimated Marginal Means with same THI (Upper limit–lower limit)	2.37 ± 0.01 (2.34–2.39)	2.41 ± 0.01 (2.39–2.43)	0.004
Milking frequency—Estimated Marginal Means with same DIM (Upper limit–lower limit)	-	-	0.126
Milking frequency—Estimated Marginal Means with same heads (Upper limit–lower limit)	2.36 ± 0.01 (2.34–2.39)	2.42 ± 0.01 (2.39–2.44)	0.009

^1^ Analyses were performed using an analysis of covariance (ANCOVA). Values are presented as mean ± SD for descriptive daily summaries and as estimated marginal means ± SE (95% CI) for adjusted exploratory comparisons. *p* values are shown only for adjusted exploratory comparisons. THI, temperature–humidity index; T_db_, dry-bulb temperature; RH, relative humidity; DIM, days in milk.

**Table 7:** The association of Spearman’s rank correlation and regression slopes for milk yield and milking frequency relative to THI during the soybean hulls and concentrate feed periods.

Dependent Variable	Supplement Period	THI Subset Condition	*r_s_* ^1^	*p*	*b* ^2^
Milk yield	Soybean hull	All data		0.158	
	Concentrate feed	All data	−0.605	<0.001	−0.248
		Below THI threshold (78)	-	0.093	-
		Above THI threshold (78)	−0.340	<0.001	−0.881
Milking frequency	Soybean hull	All data	−0.521	<0.001	−0.017
		Below THI threshold (69)	-	0.646	-
		Above THI threshold (69)	−0.347	<0.001	−0.020
	Concentrate feed	All data	−0.688	<0.001	−0.025
		Below THI threshold (75)	−0.191	0.010	−0.007
		Above THI threshold (75)	−0.492	<0.001	−0.053

^1^ The *r_s_* value indicates Spearman’s rank correlation. ^2^ The *b* value indicates the slope of the regression equation.

**Table 8:** Number of days from 29 March 2021 to 28 March 2023 when the THI in the barns exceeded the corresponding thresholds.

	Mean THI ≧ 69 Days/Total Days	Mean THI ≧ 75 Days/Total Days	Mean THI ≧ 78 Days/Total Days
29 March 2021 to 28 March 2022	261/365	195/365	149/365
29 March 2022 to 28 March 2023	262/365	182/365	131/365
Aggregate Total	523/730	377/730	280/730
Overall Percentage	71.64%	51.64%	38.36%

THI, temperature–humidity index.

## Data Availability

The data are not publicly available due to restrictions associated with the confidentiality of farm-specific information.

## References

[1] Jacobs J., Siegford J. (2012). Invited review: The impact of automatic milking systems on dairy cow management, behavior, health, and welfare. J. Dairy Sci..

[2] Speroni M., Pirlo G., Lolli S. (2006). Effect of automatic milking systems on milk yield in a hot environment. J. Dairy Sci..

[3] Melin M., Svennersten-Sjaunja K., Wiktorsson H. (2005). Feeding patterns and performance of cows in controlled cow traffic in automatic milking systems. J. Dairy Sci..

[4] Soberon F., Ryan C., Nydam D., Galton D., Overton T. (2011). The effects of increased milking frequency during early lactation on milk yield and milk composition on commercial dairy farms. J. Dairy Sci..

[5] Dikmen S., Hansen P. (2009). Is the temperature-humidity index the best indicator of heat stress in lactating dairy cows in a subtropical environment?. J. Dairy Sci..

[6] Hahn G.L. (1999). Dynamic responses of cattle to thermal heat loads. J. Anim. Sci..

[7] West J.W. (2003). Effects of heat-stress on production in dairy cattle. J. Dairy Sci..

[8] Spiers D., Spain J., Sampson J., Rhoads R. (2004). Use of physiological parameters to predict milk yield and feed intake in heat-stressed dairy cows. J. Therm. Biol..

[9] Becker C., Collier R., Stone A. (2020). Invited review: Physiological and behavioral effects of heat stress in dairy cows. J. Dairy Sci..

[10] Heinicke J., Hoffmann G., Ammon C., Amon B., Amon T. (2018). Effects of the daily heat load duration exceeding determined heat load thresholds on activity traits of lactating dairy cows. J. Therm. Biol..

[11] Wildridge A.M., Thomson P.C., Garcia S.C., John A.J., Jongman E.C., Clark C.E., Kerrisk K.L. (2018). The effect of temperature-humidity index on milk yield and milking frequency of dairy cows in pasture-based automatic milking systems. J. Dairy Sci..

[12] Li G., Chen S., Chen J., Peng D., Gu X. (2020). Predicting rectal temperature and respiration rate responses in lactating dairy cows exposed to heat stress. J. Dairy Sci..

[13] Bouraoui R., Lahmar M., Majdoub A., Belyea R. (2002). The relationship of temperature-humidity index with milk production of dairy cows in a Mediterranean climate. Anim. Res..

[14] Pinto S., Hoffmann G., Ammon C., Amon T. (2020). Critical THI thresholds based on the physiological parameters of lactating dairy cows. J. Therm. Biol..

[15] Johansson B., Uvnäs-Moberg K., Knight C.H., Svennersten-Sjaunja K. (1999). Effect of feeding before, during and after milking on milk production and the hormones oxytocin, prolactin, gastrin and somatostatin. J. Dairy Res..

[16] Prescott N., Mottram T., Webster A. (1998). Relative motivations of dairy cows to be milked or fed in a Y-maze and an automatic milking system. Appl. Anim. Behav. Sci..

[17] Schwanke A., Dancy K., Didry T., Penner G., DeVries T. (2019). Effects of concentrate location on the behavior and production of dairy cows milked in a free-traffic automated milking system. J. Dairy Sci..

[18] National Research Council (1971). A Guide to Environmental Research on Animals.

[19] Thom E.C. (1959). The discomfort index. Weatherwise.

[20] Akaike H. (1974). A new look at the statistical model identification. IEEE Trans. Autom. Control.

[21] Castro A., Pereira J., Amiama C., Bueno J. (2012). Estimating efficiency in automatic milking systems. J. Dairy Sci..

[22] King M., Pajor E., LeBlanc S., DeVries T. (2016). Associations of herd-level housing, management, and lameness prevalence with productivity and cow behavior in herds with automated milking systems. J. Dairy Sci..

[23] Halachmi I., Adan I., Van Der Wal J., Van Beek P., Heesterbeek J. (2003). Designing the optimal robotic milking barn by applying a queuing network approach. Agric. Syst..

[24] Halachmi I. (2009). Simulating the hierarchical order and cow queue length in an automatic milking system. Biosyst. Eng..

[25] Erdman R.A., Varner M. (1995). Fixed yield responses to increased milking frequency. J. Dairy Sci..

[26] Simões Filho L.M., Lopes M.A., Brito S.C., Rossi G., Conti L., Barbari M. (2020). Robotic milking of dairy cows: A review. Semin. Cienc. Agrar..

[27] Lessire F., Moula N., Hornick J.-L., Dufrasne I. (2020). Systematic review and meta-analysis: Identification of factors influencing milking frequency of cows in automatic milking systems combined with grazing. Animals.

[28] Veysset P., Wallet P., Prugnard E. Automatic milking systems: Characterising the farms equipped with AMS, impact and economic simulations. Proceedings of the Conférence Internationale: Physiological and Technical Aspects of Machine Milking.

[29] Bach A., Devant M., Igleasias C., Ferrer A. (2009). Forced traffic in automatic milking systems effectively reduces the need to get cows, but alters eating behavior and does not improve milk yield of dairy cattle. J. Dairy Sci..

[30] Berman A., Folman Y., Kaim M., Mamen M., Herz Z., Wolfenson D., Arieli A., Graber Y. (1985). Upper critical temperatures and forced ventilation effects for high-yielding dairy cows in a subtropical climate. J. Dairy Sci..

[31] Kadzere C.T., Murphy M., Silanikove N., Maltz E. (2002). Heat stress in lactating dairy cows: A review. Livest. Prod. Sci..

[32] Pilatti J.A., Vieira F.M.C., Rankrape F., Vismara E.S. (2019). Diurnal behaviors and herd characteristics of dairy cows housed in a compost-bedded pack barn system under hot and humid conditions. Animal.

[33] Allen J.D., Hall L.W., Collier R.J., Smith J.F. (2015). Effect of core body temperature, time of day, and climate conditions on behavioral patterns of lactating dairy cows experiencing mild to moderate heat stress. J. Dairy Sci..

[34] Chen J.M., Schütz K.E., Tucker C.B. (2013). Dairy cows use and prefer feed bunks fitted with sprinklers. J. Dairy Sci..

[35] Kovacs L., Kezer F.L., Poti P., Boros N., Nagy K. (2020). Short communication: Upper critical temperature-humidity index for dairy calves based on physiological stress variables. J. Dairy Sci..

[36] Gaughan J., Mader T.L., Holt S., Lisle A. (2008). A new heat load index for feedlot cattle. J. Anim. Sci..

[37] Zhou M., Aarnink A., Huynh T., van Dixhoorn I., Koerkamp P.G. (2022). Effects of increasing air temperature on physiological and productive responses of dairy cows at different relative humidity and air velocity levels. J. Dairy Sci..

[38] Mader T.L., Johnson L.J., Gaughan J. (2010). A comprehensive index for assessing environmental stress in animals. J. Anim. Sci..

[39] Shiao T., Yang D., Lee H., Hsieh C., Lee S., Lee C. (2009). Feasibility assessment of heat stress alleviation for Holstein lactating cows by water-padding barn in Taiwan. I: Barn environment and cows’ physiological responses. J. Chin. Soc. Anim. Sci..

[40] Shiao T., Yang D., Lee H., Hsieh C., Lee S., Lee C. (2009). Feasibility assessment of heat stress alleviation for Holstein lactating cows by water-padding barn in Taiwan. II: Intake activity, rumen digestion and lactation performance. J. Chin. Soc. Anim. Sci..

[41] Gebremedhin K.G., Hillman P., Lee C., Collier R., Willard S., Arthington J., Brown-Brandl T. (2008). Sweating rates of dairy cows and beef heifers in hot conditions. Trans. ASABE.

[42] Talukder S., Qiu D., Thomson P., Cheng L., Cullen B. (2023). Impact of heat stress on dairy cow rumination, milking frequency, milk yield and quality in a pasture-based automatic milking system. Anim. Prod. Sci..

[43] Morales-Piñeyrúa J.T., Damián J.P., Banchero G., SantAnna A.C. (2022). The effects of heat stress on milk production and the grazing behavior of dairy Holstein cows milked by an automatic milking system. J. Anim. Sci..

[44] Armstrong D. (1994). Heat stress interaction with shade and cooling. J. Dairy Sci..

[45] Gálik R., Lüttmerding G., Boďo Š., Knížková I., Kunc P. (2021). Impact of heat stress on selected parameters of robotic milking. Animals.

[46] Lee C.J., Fan G.J., Chang C.T., Lin Y.J. (2024). Assessing the Goodness of Fit of Different Temperature-humidity Index Models on Milk Yield and Milking Frequency in Auto-milking Systems in Hot and Humid Environment. J. Agric. For..

[47] Hammami H., Bormann J., M’hamdi N., Montaldo H.H., Gengler N. (2013). Evaluation of heat stress effects on production traits and somatic cell score of Holsteins in a temperate environment. J. Dairy Sci..

[48] Spiers D., Spain J., Ellersieck M., Lucy M. (2018). Strategic application of convective cooling to maximize the thermal gradient and reduce heat stress response in dairy cows. J. Dairy Sci..

[49] National Academies of Sciences, Engineering, and Medicine (2021). Nutrient Requirements of Dairy Cattle: Eighth Revised Edition.

[50] Madsen J., Weisbjerg M.R., Hvelplund T. (2010). Concentrate composition for automatic milking systems—Effect on milking frequency. Livest. Sci..

[51] Halachmi I., Shoshani E., Solomon R., Maltz E., Miron J. (2009). Feeding soyhulls to high-yielding dairy cows increased milk production, but not milking frequency, in an automatic milking system. J. Dairy Sci..

[52] Bach A., Cabrera V. (2017). Robotic milking: Feeding strategies and economic returns. J. Dairy Sci..

[53] Rousing T., Badsberg J.H., Klaas I.C., Hindhede J., Sørensen J.T. (2006). The association between fetching for milking and dairy cows’ behaviour at milking, and avoidance of human approach—An on-farm study in herds with automatic milking systems. Livest. Sci..

[54] Drach U., Halachmi I., Pnini T., Izhaki I., Degani A. (2017). Automatic herding reduces labour and increases milking frequency in robotic milking. Biosyst. Eng..

[55] Bava L., Tamburini A., Penati C., Riva E., Mattachini G., Provolo G., Sandrucci A. (2012). Effects of feeding frequency and environmental conditions on dry matter intake, milk yield and behaviour of dairy cows milked in conventional or automatic milking systems. Ital. J. Anim. Sci..

[56] IPCC (2021). Climate Change 2021: The Physical Science Basis. Contribution of Working Group I to the Sixth Assessment Report of the Intergovernmental Panel on Climate Change.

[57] Central Meteorological Bureau (2021). Excerpts of Scientific Highlights from the IPCC Sixth Assessment Report on Climate Change and Taiwan’s Climate Change Assessment Update Report; Taiwan Climate Change Estimation Information and Adaptation Knowledge Platform.

[58] Provolo G., Riva E. (2009). One year study of lying and standing behaviour of dairy cows in a frestall barn in Italy. J. Agric. Eng..

